# “This Is How I Give Back”: Long-Term Survivors on Legacy and HIV Cure Research at the End of Life—A Qualitative Inquiry in the United States

**DOI:** 10.3390/idr17040078

**Published:** 2025-07-04

**Authors:** Ali Ahmed, Jeff Taylor, Whitney Tran, Simran Swaitch, Samuel O. Ndukwe, Rachel Lau, Kris H. Oliveira, Stephanie Solso, Cheryl Dullano, Andy Kaytes, Patricia K. Riggs, Robert Deiss, Sara Gianella, Karine Dubé

**Affiliations:** 1 Division of Infectious Diseases and Global Public Health, School of Medicine, University of California San Diego (UCSD), 9500 Gilman Drive, La Jolla, CA 92093, USA; aliahmed@health.ucsd.edu (A.A.); wht001@health.ucsd.edu (W.T.); sswaitch@ucsd.edu (S.S.); sndukwe@health.ucsd.edu (S.O.N.); rmlau@ucsd.edu (R.L.); ssolso@health.ucsd.edu (S.S.); cdullano@health.ucsd.edu (C.D.); pariggs@health.ucsd.edu (P.K.R.); rgdeiss@health.ucsd.edu (R.D.); gianella@health.ucsd.edu (S.G.); 2 HIV+ Aging Research Project-Palm Springs, Palm Springs, CA 92264, USA; jefftaylorps@gmail.com; 3 Reversing Immune Dysfunction for HIV-1 (RID-HIV) Eradication Martin Delaney Collaboratory Community Engagement Coordination and Community Advisory Board, AVRC, San Diego, CA 92037, USA; andykaytes@gmail.com; 4 Departamento de Infectologia e Medicina Tropical, Faculdade de Medicina da Universidade de São Paulo (FMUSP), São Paulo 05403-000, Brazil; kris.h.oliveira@gmail.com

**Keywords:** HIV cure research, people with HIV, end of life, socio-behavioral research, the United States

## Abstract

**Background/Objectives**: End-of-life (EOL) HIV cure research, which studies HIV persistence through pre- and post-mortem tissue collection, has focused primarily on people living with HIV (PLWH) with a prognosis of six months or less. However, the perspectives of long-term survivors (LTS) diagnosed before the advent of effective antiretroviral treatment (ART) remain underexplored. Understanding their motivations and concerns about EOL cure research is essential for creating inclusive and ethical research frameworks. **Methods**: Between 2023 and 2024, we conducted in-depth qualitative interviews with 16 PLWH aged 60 and older from diverse backgrounds across the United States, recruited through community-based organizations and HIV networks. We used inductive thematic analysis to explore LTS’ perspectives on EOL HIV research. **Results**: Participants included cisgender men (56.25%) and women (43.75%) with diverse racial identities. While participants supported EOL HIV cure research, their willingness to participate varied, influenced by awareness, logistics, and ethical concerns. Altruism-motivated participation, but misconceptions about procedures and concerns over bodily integrity represented potential barriers. Some viewed blood draws and leukaphereses as routine, while others expressed hesitancy with biopsies and post-mortem tissue retrieval. HIV stigma, historical mistrust, and cultural beliefs also played a role in willingness to participate. LTS emphasized the need for decentralized research sites, travel support, and financial safeguards. **Conclusions**: To include LTS in EOL HIV cure research, a community-driven approach is needed, focusing on clear communication, ethical considerations, logistical support, and linkages to EOL care. Addressing misconceptions and building trust, particularly within groups traditionally underrepresented in research, is essential to expanding participation.

## 1. Introduction

Treatment advances have transformed human immunodeficiency virus (HIV) from a terminal diagnosis to a chronic, manageable condition, but the search for an HIV cure remains an enduring challenge [1]. Although antiretroviral therapy (ART) effectively suppresses viral replication, it does not eliminate HIV from the body, requiring lifelong medication adherence and exposing people living with HIV (PLWH) to potential long-term toxicities [2,3]. A critical barrier to achieving a cure is the persistence of HIV in latent reservoirs, which can only be fully characterized through direct sampling of deep tissues [1]. End-of-life (EOL) HIV cure research provides a rare opportunity to study these reservoirs, as altruistic PLWH at the EOL are willing to donate biological samples both before and after death, yielding tremendous scientific insights otherwise unattainable in living individuals [4].

The Last Gift study at the University of California San Diego (UCSD) represents a novel approach in providing a framework for PLWH at the EOL to engage in HIV cure-oriented research, consistent with initiatives set forward by the National Institute of Allergy and Infectious Diseases (NIAID) and the National Institute of Mental Health (NIMH) [5]. While most HIV cure trials are limited to otherwise healthy volunteers [6], the Last Gift observational cohort enrolls PLWH with a non-AIDS-related terminal illness and a prognosis of six months or less, or a chronic illness and multiple co-morbidities associated with a five-year mortality of greater than 50% [5,7]. Participants undergo routine blood draws and optional biopsies during life, and upon death, a rapid research autopsy is conducted within six hours to obtain tissue samples from key anatomical sites of HIV persistence [5]. This approach allows researchers to study viral reservoirs before cellular and viral degradation occurs, generating crucial data to inform the design of future HIV cure strategies [8].

With Last Gift biospecimens now being shared with research laboratories across the United States (U.S.), ensuring ongoing community engagement and social acceptance of EOL HIV cure research is critical [9]. While prior socio-behavioral research studies conducted in Southern California have demonstrated broad support for this work [10], social acceptance is not static; it must be continuously reassessed as the field evolves and new research findings emerge. The perspectives of long-term survivors (LTS) of HIV, especially those who acquired HIV before the advent of combination ART in 1996, are crucial in shaping EOL HIV cure research [11]. Many LTS have endured decades of HIV-related stigma, early ART toxicities, and trauma, positioning them as key participants in conversations about EOL HIV cure research [6]. Their lived experiences provide valuable insights into the social, ethical, and logistical considerations of EOL HIV cure research participation.

Despite the importance of diverse LTS’ perspectives, discussions surrounding EOL HIV cure research have largely been confined to PLWH already engaged with and connected to research centers across the U.S. [9]. To ensure that these EOL studies reflect the needs and values of the broader HIV community, it is crucial to engage LTS from varied geographic, racial, ethnic, and socioeconomic backgrounds, including those in regions such as the South and rural areas [10,12]. Doing so can help address critical questions about research equity, access, and trustworthiness. Understanding how different communities perceive EOL HIV cure research, including concerns related to medical mistrust, religious and cultural considerations, and logistical barriers, is key to designing protocols that are both inclusive and ethically robust [13].

As EOL HIV cure research evolves and expands, so too must the social and ethical considerations it entails [8]. The sustainability and credibility of EOL HIV cure research depend on ongoing dialogue with the community of PLWH rather than assuming that prior findings on acceptability remain indefinitely relevant. The present study sought to engage LTS of HIV across the entire U.S. to understand their perceptions on HIV cure research at the EOL. Through in-depth interviews (IDIs), we aim to identify facilitators and barriers to participation, assess awareness and perceptions, and explore how best to integrate EOL research into the broader HIV cure research agenda in a way that is transparent, inclusive, and ethically sound. By centering the voices of aging PLWH, particularly LTS, this work can help inform the practices for engaging communities in EOL HIV cure research while upholding ethical and community-engaged research standards. The insights gathered from these interviews can help shape the future of EOL HIV cure research, ensuring that it reflects the needs, concerns, and values of those most affected by HIV.

## 2. Methods

### 2.1. Study Design

We chose a qualitative approach to explore the nuanced perspectives of aging PLWH on EOL HIV cure research, addressing ethical, social, and logistical considerations that quantitative methods may overlook [14]. Through IDIs, participants can share their views in their own words, allowing scientists to fully examine concerns about medical mistrust, cultural and religious considerations, and barriers or facilitators to participation. Qualitative approaches also enable the engagement of underrepresented voices, particularly those outside traditional research centers, and can help us understand how nationwide attitudes toward EOL research evolve.

### 2.2. Study Setting and Participants

This study was a subset of a larger U.S.-based project aimed at understanding the perspectives of LTS of HIV on cure research. For the EOL research component, we excluded individuals unable or unwilling to discuss EOL HIV cure research, those unable to provide informed consent, and non–English speakers (since interviews were conducted in English). We recruited participants through community partners affiliated with three Martin Delaney Collaboratories for HIV Cure Research: (1) Delaney AIDS Research Enterprise (DARE), (2) Reversing Immune Dysfunction for HIV-1 Eradication (RID-HIV), and (3) CRISPR for Cure. We also recruited LTS from our three community-based organizations (CBOs), referred to as (1) AIDS Action Baltimore, (2) HIV + Aging Research Project—Palm Springs (HARP-PS), and (3) the Reunion Project, along with other CBOs focused on HIV and aging, including NMAC (formerly the National Minority AIDS Council), The Well Project, and the San Francisco AIDS Foundation (SFAF). Several individuals referred by community partners agreed to participate; we did not systematically document refusals or reasons for non-participation.

To further enhance diversity, we distributed an institutional review board (IRB)-approved flyer and asked investigators and clinicians specializing in HIV and aging to refer LTS of HIV from varied backgrounds. This purposive, non-probabilistic sampling strategy captured perspectives from a broad range of participants. Participants understood that participation in this study did not imply participation in future EOL clinical research. By the 16th interview, we reached data saturation, as no new themes emerged, confirming the depth and breadth of perspectives gathered [15].

### 2.3. Interview Guide

We collaborated with LTS of HIV and community advisors to develop the semi-structured interview guide (Table 1). LTS advisors then reviewed the guide for clarity and relevance. We did not conduct formal pilot testing. This semi-structured guide covered key areas, including general knowledge and perceptions of EOL HIV cure research, motivations and barriers to participation, and ethical considerations such as medical trust, cultural and religious beliefs, and research equity. We also explored willingness to undergo invasive procedures, strategies for engaging underrepresented groups, and potential regulatory and policy considerations in EOL HIV cure research. Open-ended questions encouraged participants to share nuanced perspectives, providing a deeper understanding of their concerns and priorities related to this type of research.

### 2.4. Data Collection

The principal investigator (K.D.) provided participants with an IRB-approved informed consent form, a demographic questionnaire, and an interview guide upon scheduling their interviews. Two research team members (K.D. and J.T.) conducted and recorded the interviews using a HIPAA-compliant virtual conferencing platform (Zoom). Interviews lasted approximately 30–60 min and were held in English via audio and/or video teleconference, based on participants’ preferences, ensuring privacy and security. Each participant received a USD 50 Visa gift card as compensation, either as a virtual card or a physical card sent by mail, based on their preferences.

### 2.5. Data Analysis

Audio files of each interview were saved to a secure drive, with access restricted to research team members, and then uploaded to the website of an encrypted transcription service for verbatim transcription. Subsequently, research assistants (W.T., S.S.) carefully reviewed each transcript for de-identification, completeness, and accuracy, resolving discrepancies with the audio source record. Participants did not review the transcripts or the findings. Given the qualitative approach of this study, we used conventional thematic analysis [16] with inductive reasoning to analyze the data.

All de-identified responses were collated into a master document, in which they were categorized by question and prepared for manual coding. We did not apply for a pre-existing coding scheme due to the novelty of this research. The primary coder, a post-doctoral researcher (A.A.), developed the initial codebook and extracted relevant quotes. The principal investigator (K.D.) assumed the role of the secondary coder, reviewing and refining the initial codes, and organizing the quotes into a tabular format. Both researchers utilized an inductive approach to identify emergent themes and resolved discrepancies through consensus during bi-weekly virtual meetings. We assessed theme salience qualitatively based on the recurrence of themes across interviews, the depth of discussion, and consensus among coders. After reviewing the data, the research team concluded that thematic saturation had been achieved [16].

We used OpenAI’s ChatGPT (GPT-4, OpenAI Inc., San Francisco, CA, USA; accessed on 27 February 2025) to support language editing during manuscript preparation. Specifically, this tool was used to improve sentence clarity, grammar, and overall readability. No content generation, data analysis, or interpretation of results was conducted by the AI. All thematic coding and interpretation were performed by the human research team, with AI involvement limited strictly to linguistic refinement under direct human supervision.

Illustrative quotes exemplifying major themes are presented in the results section, with additional quotes provided in Supplementary Table S1.

### 2.6. Ethics Statement

We conducted this study in compliance with all relevant guidelines and regulations, including the U.S. Code of Federal Regulations and the Declaration of Helsinki. The UCSD IRB approved this study (IRB #808882). Prior to their interview, participants received a copy of the informed consent form. Their verbal consent was then recorded during their interview. To protect confidentiality, we de-identified all study-related documents and transcripts and destroyed the audio files following the quality verification of transcription.

## 3. Results

### 3.1. Participants’ Characteristics

This study included 16 PLWH who identified as LTS with a mean age of 68.4 years (range: 60–82). Most identified as cisgender men (56.25%) and cisgender women (43.75%), with racial identities including White/Caucasian (56.25%), Black/African (25%), Asian (12.5%), and mixed race (6.25%). Over half (56.25%) were college graduates, and participants were geographically distributed across the West (50%), South (31.25%), Northeast (12.5%), and Midwest (6.25%) regions of the U.S. On average, participants had 27.13 years (SD: 10.46) of HIV-related advocacy experience and 7.34 years (SD: 9.78) in HIV cure research-related advocacy (Table 2).

### 3.2. Structure of Themes

The results are structured around key topics. Briefly, *Awareness and Perceptions of HIV Cure Research at the EOL* highlights participants’ knowledge and attitudes, while *Motivations and Barriers for Participation* explore factors influencing engagement, including altruism and logistical concerns. *Willingness to Undergo Invasive Study Procedures* examines participants’ perspectives on medical procedures near or at the EOL. *Outreach, Justice, Equity, and Trustworthiness* addresses the need for inclusive recruitment and trust-building. *Religious and Cultural Considerations* explore how beliefs may shape willingness to participate, and *Regulatory and Policy Considerations* focus on ethical oversight, hospice integration, and participant autonomy.

#### 3.2.1. Awareness and Perceptions of HIV Cure Research at the End of Life

##### Awareness of HIV Cure Research at the EOL

We first inquired about participants’ awareness of EOL HIV cure research. LTS described varying levels of familiarity with this research, ranging from general knowledge to personal engagement. Many specifically referenced the UCSD Last Gift study or similar rapid autopsy initiatives in the cancer field. These programs were recognized for advancing basic sciences, enhancing understanding of HIV reservoirs, and contributing to HIV cure research.

*So yes, I’ve heard of it. They are doing some research with bits of what they gleaned from the autopsy… I personally think it’s a wonderful thing. This is how I give back, I even love the name Last Gift… it’s a real validation… the last gift I can give.*

—P01, Cisgender Man, White

However, some participants were unaware of EOL HIV cure research until they came across specific community outreach efforts. Even within LTS networks, awareness remained low, with few healthcare providers discussing this research option. Participants stressed the need for broader dissemination through diverse platforms.

*This is the first time I heard about end-of-life HIV research. Usually, the research is about medication and stuff like that. But I like your research study because it’s about how to age properly and how to die with dignity.*

—P08, Cisgender Women, Asian

Although we did not systematically probe prior awareness, only a few participants spontaneously reported unfamiliarity; after a brief explanation, their motivations and concerns mirrored those of participants already familiar with these concepts. Likewise, LTS suggested community-based education efforts, including intergenerational discussions and forums at lesbian, gay, bisexual, transgender, and queer or questioning (LGBTQ+) centers, to foster engagement. Some expressed frustration that such research was limited to specific institutions like UCSD, calling for expansion of similar programs across the entire U.S.

*This is a program that should be expanded, not just limited to one institution, but available to people with HIV at the end of life everywhere in the United States.*

—P10, Cisgender Woman, Black

Community ambassadors were seen as key to raising awareness and ensuring diverse participation in EOL HIV cure research. Participants noted that direct outreach and patient engagement could increase interest. However, misconceptions about EOL research, particularly around its timing and procedures, were cited as potential barriers to participation. Some associated it only with imminent death, while others feared invasive procedures.

*I always think of ‘end of life’ as lying in bed dying. But I think what you mean is more like approaching the end of life. That’s different.*

—P02, Cisgender Man, White

##### Perceptions of HIV Cure Research at the EOL

Many participants viewed research at the EOL as essential for advancing science and addressing long-standing barriers in HIV care. They emphasized that critical insights, such as understanding HIV reservoirs and drug penetration into tissues, can only be obtained from post-mortem studies. Participants, who have lived with HIV for decades and taken various medications, viewed their peers and themselves as uniquely valuable contributors. Many supported EOL research as a meaningful way to give back to the HIV community and validate their lived experiences.

*I think the research desperately needs to be done. Because of the history of HIV and stigmatization, there’s still hesitation to deal with these issues… But if it’s advancing towards a cure, I think it’s great.*

—P02, Cisgender Man, White

*It absolutely should be done… There are certain kinds of research that can only ethically be done at the end of life, such as studying drug kinetics in the brain. This research could provide very valuable results for everybody with HIV.*

—P03, Cisgender Man, White

*I’ve heard about stopping treatment and looking for reservoirs… You really need to figure that out because we might not get anywhere until we do.*

—P05, Cisgender Woman, Mixed race

*Absolutely. I mean, that’s a unique and special way to obtain answers that you can’t get during life. Finding out where it [HIV]’s hiding could be really helpful information, not just for HIV, but for other areas of science too.*

—P14, Cisgender Woman, White

Many participants expressed optimism that EOL research would help contribute to an eventual cure, even if it would not directly benefit participants.

*I think there are more people like me who would, given the opportunity, really participate. There’s so much opportunity for furthering research, giving the researchers materials to work with is simple, and it’s something I can do.*

—P13, Cisgender Man, White

#### 3.2.2. Motivations and Barriers for Participation in HIV Cure Research at the EOL

Participants’ willingness to contribute to EOL HIV cure research was largely driven by altruism, a desire to leave a meaningful legacy, and a need for connection and dignity. However, these themes were in tension with fears of discomfort and the unknown, societal and family pressures, and logistical concerns such as burial costs and research-related delays.

##### Altruism Versus Fear of Discomfort and the Unknown

For many LTS, altruism was the primary motivator for their interest in EOL HIV cure research options. They viewed participation as a way to ensure future generations do not endure the hardships they have faced and to advance scientific understanding of HIV. Some framed their motivation as a deep personal mission to leave behind a legacy of hope.

*First and foremost, it is always altruism. At the end of life, I don’t think that I’m going to get much personal benefit, other than an altruistic one.*

—P01, Cisgender Man, White

*If my dead body can make it so that no one else has to go through what I and many of my friends have gone through over the last 40 years, take it and do what you need to do.*

—P07, Cisgender Man, White

*I realized that here’s a unique chance to provide really valuable data that could help the entire field of HIV and immunology research. That’s a great appeal to me personally, and I would imagine many others.*

—P03, Cisgender Man, White

Yet, despite this strong sense of altruism, fear remained a potential barrier. For some, it was the fear of procedure side effects or discomfort during the research process. Others were hesitant due to the unknown—what would happen to their bodies, whether procedures would be painful, and how they would feel in their final moments.

*Fear. I mean, fear is the first big one that pops into my head… fear of side effects, fear of discomfort.*

—P01, Cisgender Man, White

##### Desire for Connection and Dignity Versus Societal Pressure

Beyond scientific contributions, many participants viewed research participation as a means to combat loneliness and discover purpose at the EOL. Some framed it as an avenue to avoid isolation, emphasizing that even the structure of this study could provide a meaningful connection to others.

*We can’t forget that there are people who are alone. And it might not be the greatest form of personal contact, but it’s personal contact. I’d rather not sit at home alone, afraid to talk to people about my health status.*

—P01, Cisgender Man, White

Some connected their desire to participate with a sense of dignity and control over their legacy, particularly LTS who had suffered the loss of loved ones and endured the worst of the HIV epidemic in the early days.

*Those of us who’ve seen this disease for a long time, who saw the tragedy and the lives claimed, I think we would really like to contribute in some meaningful way.*

—P14, Cisgender Woman, White

However, for some, the desire to participate clashed with social pressures from loved ones. Some worried their next-of-kin/loved ones would not accept their decision, while others noted pressure to be present with their loved ones towards the EOL rather than focus on research participation.

*Family might be a deterrent. They may want their loved ones to be with them instead of participating in research.*

—P09, Cisgender Man, White

For others, concerns extended beyond immediate family pressure. They worried about how research participation might affect their grieving loved ones, particularly regarding how quickly their remains would be returned.

*They think it’s going to be hard on the family left behind.*

—P08, Cisgender Woman, Asian

##### Practical Considerations Versus Logistical Barriers

For some, burial and cremation costs created significant stress at the EOL. This made EOL research participation more appealing as it may help alleviate costs related to EOL. Participants viewed covering EOL-related expenses, such as cremation costs, as a major relief, influencing their willingness to engage.

*I think that covering death and cremation relieves some anxieties that people might have at end of life.*

—P04, Cisgender Man, White

*Most people don’t have the money for a funeral, and I think taking care of cremation costs would be a significant motivation.*

—P05, Cisgender Woman, Mixed race

In addition, transportation and logistical challenges created obstacles for participation. Some participants noted that travelling to study sites or covering travel costs for themselves and caregivers was difficult and could prevent engagement in EOL research. Concerns about burial delays also led to some hesitance, due to confusion regarding the length of time their body would be held for research. Misconceptions about post-mortem timelines contributed to uncertainty, making some reluctant to participate.

#### 3.2.3. Willingness to Undergo Invasive Study Procedures near or at the EOL

Participants expressed a range of perceptions about undergoing invasive study procedures near or at EOL, such as blood draws, leukaphereses, and gut and lymph node biopsies. Understanding these perceptions is crucial for assessing the feasibility of research that compares HIV reservoirs before and after death, as such studies rely on participants’ willingness to undergo these procedures. Participants reported that these medical procedures have been a routine part of their care. As a result, most did not perceive procedures like blood draws or leukaphereses as particularly invasive.

*We have been poked and prodded, and, you know, spinal taps and apheresis for years… I don’t consider any of these invasive.*

—P01, Cisgender Man, White

*By the time that we’re close to the end, we’ve all gone through similar studies… I don’t know that [if] that’s particularly more invasive than things many of us have gone through in our lifetime.*

—P14, Cisgender Woman, White

Participants acknowledged that perceptions of study procedure invasiveness may vary across populations. Concerns about pain and discomfort were particularly significant for those already managing chronic pain or terminal illnesses. Many emphasized that the decision to undergo such procedures would depend on an individual’s health status and capacity to tolerate these interventions. Some participants emphasized the need for clear, visual explanations to ensure participants fully understand invasive procedures. While some participants mentioned diagrams to enhance understanding, others suggested that visual aids, such as videos, could also be useful.

#### 3.2.4. Outreach, Justice, Equity, and Trustworthiness Considerations for HIV Cure Research at the EOL

Participants noted that expanding access to EOL HIV cure research requires community-driven outreach that reflects the diverse identities, experiences, and geographic needs of PLWH across the U.S. Participants stressed the need for transparency and emphasized the importance of addressing their doubts, emotional well-being, accessibility, historical mistrust, and cultural responsiveness to ensure that research is inclusive, ethical, and representative.

##### Ensuring Transparency and Informed Decision-Making

Participants suggested providing clear, detailed, and honest information about this study, including the scope of procedures, potential benefits, and possible discomforts, as essential in fostering trust and encouraging participation in EOL research. Many stressed that fully transparent informed consent, including visual aids and discussions with medical professionals, would help alleviate fears and uncertainty near the EOL. Additionally, understanding that participation is voluntary and that they could withdraw at any time was seen as crucial in their decision-making process.

*I have to be… competent to be making the correct decision. They have to be given a well-written… informed consent… as long as participants are deemed competent… and there are well-thought-out guardrails in place, I have no problem with this.*

—P01, Cisgender Man, White

*Facts, truths, nothing hidden. Honesty is the best policy.*

—P16, Cisgender Woman, Black

Participants also emphasized the importance of engaging next-of-kin/loved ones and caregivers in these discussions, particularly for individuals making EOL research decisions.

##### Addressing Mental Health and Social Support Needs

Many PLWH emphasized that EOL research participation can be emotionally complex, particularly for those who are socially isolated, estranged from family, or experiencing cognitive decline. They called for integrated mental health resources, including trauma-informed counseling, memory care, and social support networks, to help individuals process their decisions and navigate research participation.

*One of our priorities should be mental health. Some of us may be alone. We also have to think about our physical health, because not everyone has the same access to care.*

—P01, Cisgender Man, White

*Social support is really important. They need somebody they can talk to, or even a hotline, or even someone to talk to the families, to ease their mind.*

—P08, Cisgender Woman, Asian

##### Peer and Community Support

To enhance support in EOL HIV cure research, participants recommended a structured peer support system where individuals not at EOL could serve as study buddies for those enrolled in this research. Many LTS were familiar with supporting peers from the early days of HIV/AIDS. These buddies could be LTS or experienced study participants who provide emotional support, assist with navigating study procedures, accompany participants to appointments, and help address logistical challenges. Participants suggested this approach would build existing community-based support models and ensure that participants are not going through the process alone.

*We used to do buddy systems back in HIV days and AIDS days. Why not link someone in the study with someone in the study? Go through it together.*

—P02, Cisgender Man, White

Participants viewed these community-based partnerships as essential to fostering connection, trust, and engagement in EOL research.

##### Expanding Outreach Beyond Traditional Research Centers

Participants emphasized the importance of expanding EOL research opportunities beyond major metropolitan centers to ensure broader accessibility and inclusivity. Many noted that current recruitment efforts often reach a limited demographic, primarily older, white, well-educated individuals, highlighting the need for proactive engagement in diverse communities. Bringing HIV cure research opportunities to local settings—such as community centers, pride events, and rural areas—while destigmatizing the processes of death and dying, could help ensure more equitable participation.

*Even though I live in a big city, I only heard about this research recently. Why isn’t this information reaching more people?*

—P07, Cisgender Man, White

*You have to find a gatekeeper who knows their community and can help educate them [others]. That’s important.*

—P16, Cisgender Woman, Black

##### Reducing Stigma Through Representation and Inclusive Research

Participants identified stigma surrounding both HIV and EOL decision-making as a major barrier to research participation. Participants encouraged collaborations with trusted community leaders and advocates to reduce stigma and normalize conversations around HIV and EOL research.

*In my community, stigma is still there… I represent my people, and if you need someone to speak for women, HIV, or Asians, I will.*

—P08, Cisgender Woman, Asian

Participants emphasized that inclusive and diverse research teams play a critical role in creating culturally safe and affirming spaces for these discussions.

*If you’re saying you’re inclusive, but you look around and don’t see diversity, it’s going to be difficult to believe.*

—P06, Cisgender Man, Black

##### Tailored Communication and Accessibility

Participants highlighted the importance of competent and inclusive communication in research outreach. Language barriers and a lack of tailored messaging created hurdles to participation. They emphasized that accessible study materials in multiple languages and research staff who reflect the linguistic backgrounds of participants are key to building trust.

*Everything should be translated, at least into Spanish and ASL [American Sign Language]. We should not have to work to understand what is being said or be ashamed or afraid to say, ‘I don’t understand.’ A study team should not only look like me but also speak my language.*

—P01, Cisgender Man, White

By prioritizing linguistic accessibility, awareness, and visible diversity in research teams, studies can cultivate a more inclusive, stigma-free environment that encourages participation across diverse communities.

##### Addressing Historical Distrust and Medical Mistrust

Historical mistreatment of racially and ethnically minoritized groups continues to shape distrust in research. Participants referenced past abuses, such as the U.S. Public Health Service Syphilis Study at Tuskegee, highlighting the lasting impact of systemic racism and discrimination. Many emphasized that rebuilding trust requires researchers to acknowledge these histories, engage communities directly, and ensure that research participation reflects diverse populations.

*A lot of Black folks still say, ‘I’m not going to let them make a test dummy out of me.’ It’s a lack of understanding of what research really does.*

—P10, Cisgender Woman, Black

*The research community needs to engage directly with communities and ensure treatments reflect all populations so that people trust that they will work for them.*

—P13, Cisgender Man, White

More broadly, participants described personal and collective negative experiences with healthcare, reinforcing skepticism toward research. Some recounted instances of mistreatment or inadequate care, while others noted general distrust of medical professionals and government-led initiatives.

*There are people who feel they’ve been abused by the medical community, dismissed, given less-than-adequate care. Some people are just done.*

—P07, Cisgender Man, White

*Some people develop attitudes where they feel like doctors know nothing, and they don’t trust them.*

—P05, Cisgender Woman, Mixed race

To address these concerns, participants suggested that researchers build trust by collaborating with respected community leaders, practicing full transparency, openly acknowledging past harms, and striving to be trustworthy.

#### 3.2.5. Religious and Cultural Considerations in HIV Cure Research at End of Life

Ensuring religious and cultural competency in HIV cure research at the EOL is essential for fostering inclusivity and building trust among diverse communities. Several themes emerged regarding how religion and cultural factors shape perspectives on participation in HIV cure research.

##### Religious and Spiritual Considerations

Certain religious traditions require bodies to remain intact after death, which may present a significant barrier to research participation at the EOL. This belief is particularly strong in some Jewish, Muslim, and Christian traditions, where the body must be preserved for burial or resurrection. LTS noted that addressing these concerns with religious leaders and offering alternative options may help mitigate reluctance to participate.

*I think you’re going to find a lot of resistance in the Jewish or Muslim communities because of religious laws. Jewish tradition requires burial with the whole body intact.*

—P02, Cisgender Man, White

For some, EOL research participation may raise spiritual anxieties about death, the afterlife, and religious obligations. Participants highlighted the importance of having access to religious leaders or spiritual advisors who can help navigate ethical challenges and provide counseling related to participation in medical research. Ensuring that individuals can receive religious rites, such as last rites in Catholicism, was considered essential for making research participation acceptable for some.

*I think that having somebody who can help you work through those spiritual concepts of ‘Am I committing a sin?’ or ‘Am I risking eternal damnation?’ might help ease my and my family’s fears about moving on.*

—P01, Cisgender Man, White

*I would want my spiritual leader, my priest, and stuff like that, just to know that when it gets closer, I get last rites or whatever.*

—P04, Cisgender Man, White

Engaging diverse populations in EOL, HIV cure research requires cultural competence and sensitivity to religious beliefs. Respecting cultural traditions on death and involving spiritual leaders may help bridge gaps in participation.

##### Cultural and Gender Considerations

Gender and traditional beliefs can play a significant role in how people engage with EOL research. Participants noted that some women, for example, may feel more comfortable discussing their health with women doctors, and some traditions have specific practices around death and dying that need to be considered. Moreover, attitudes toward death and dying vary significantly across cultures and can affect decisions about EOL research participation. Some communities have specific mourning traditions, while others may resist discussing death openly. Others also mentioned superstition or cultural taboos around death and tissue donation as elements that further influence these decisions.

#### 3.2.6. Regulatory and Policy Considerations of HIV Cure Research at the EOL

LTS called for regulatory improvements, policy support, and accessible hospice care to uphold dignity in EOL HIV cure research. They also stressed the need for financial security, legal protection, and equitable healthcare to prevent undue burdens on research participants.

##### Ethical Oversight and IRB Improvements

Several participants expressed concerns that IRBs, which oversee the ethics of studies, need re-examination and reform to ensure they prioritize the human aspect of research participation rather than viewing participants as data points. Participants emphasized that policies should encourage ongoing training for IRB members on ethical considerations specific to EOL research.

*Even sometimes IRBs need to be educated on a different level, they need to learn about human compassion, because I think most of them are still looking at just individuals as numbers and data but forget that those numbers and data belong to people and individuals who are real humans.*

—P16, Cisgender Woman, Black

##### Policy-Level Support

The political climate significantly affects funding and support for HIV research. Participants noted that political leadership and priorities affect the availability of resources for HIV cure research and participant support.

##### Autonomy, Hospice, and Equitable Care

Participants emphasized the need for autonomy and comprehensive hospice and palliative care when participating in EOL HIV cure research. They advocated for minimal burden and access to supportive care, stressing the importance of aging in place and ensuring comfort while participating in EOL research. Some discussed medical aid in dying (MAiD) as a personal choice and underscored the need for policies that respect individual agency. Others highlighted the necessity of integrating linkages with hospice services into EOL research, ensuring they remain HIV-informed, accessible, and stigma-free.

*I think that people should be allowed to make their own decisions. And I think that’s where the public policy fails.*

—P02, Cisgender Man, White

Participants emphasized that hospice care should not only provide comfort but also facilitate informed participation in research without compromising dignity or autonomy.

*Hospice is extremely important in allowing people a comfortable end of life. Having hospice is a natural thing and having people be familiar with hospice and willing to go for it when it’s appropriate.*

—P13, Cisgender Man, White

Ensuring clear policies and ethical safeguards will be critical in maintaining participant trust and aligning research with compassionate EOL care. Financial and housing stability were also critical concerns, particularly for those without next-of-kin support. Participants highlighted the need for affordable, HIV-informed care and noted that financial burdens, such as medical and housing costs, could deter participation.

##### End-of-Life Planning and Legal Protections

Participants stressed the need for accessible legal services to ensure individuals’ final wishes are honored in EOL HIV cure research. Living wills and power of attorney documents were seen as essential, yet access to free legal aid remains limited and should be expanded.

## 4. Discussion

To our knowledge, this is one of the first studies to assess the perspectives of diverse LTS on HIV cure research at EOL across the U.S., offering insights beyond PLWH with a terminal prognosis. This study’s strength lies in its inclusion of racially, geographically, and experientially diverse LTS, with intentional efforts to engage underrepresented groups in HIV cure research at the EOL. Participants expressed interest in contributing to HIV cure advancements, particularly in understanding HIV reservoirs; however, their willingness to participate was shaped by awareness, motivations, logistical barriers, and ethical considerations. As noted in the results, those initially unfamiliar with HIV cure or EOL research offered similar motivations and concerns once informed, underscoring the value of educational outreach. While altruism was a primary motivator, concerns such as EOL costs, logistics, and potential misconceptions about study procedures posed barriers to participation. Existing EOL HIV cure research, such as the Last Gift study, primarily enrolls PLWH near the EOL [5]. However, findings from the present study highlight the importance of expanding the scope of EOL research to include LTS who, while not at the EOL, are actively considering future participation in research. As noted, LTS expressed the need to engage in long-term planning, considering how care and research participation may align with aging in place (home-based) and medical autonomy. These findings underscore the need to expand EOL HIV cure research engagement beyond terminal diagnoses, ensuring early dialogue, clearer procedural explanations, and alignment with long-term care planning to enhance informed decision-making and accessibility [17].

A persistent theme among participants was the desire to leave behind a legacy by contributing to HIV scientific advancements. This reflects a broader pattern observed in previous studies, where altruism plays a central role in research participation among aging PLWH [4,8,10,18,19,20]. The desire to contribute stems from a historical context of activism within the HIV community, with many LTS recognizing EOL research as a final opportunity to influence the future of HIV research [8]. Despite their willingness to contribute, participants also voiced concerns surrounding the invasiveness and post-mortem uncertainties. While some viewed blood draws and leukaphereses as routine, others expressed hesitancy due to concerns about tissue retrieval and the handling of their remains. Similar hesitations have been documented in research on rapid autopsy programs in cancer studies, where fear of discomfort and the unknown can limit participation even among those who wish to contribute to science [21,22]. These concerns underscore the importance of transparent, participant-centered communication about what EOL research entails, including detailed explanations of procedures, visual aids, and discussions that foreground comfort and dignity. Some LTS participants felt reassured after learning that rapid research autopsies occur within six hours and that ashes or other EOL arrangements are handled according to their preferences; participants were also informed that, if desired, the full body can be returned for embalming and open-casket services. These details highlight the significance of clear communication and logistical support in facilitating informed decision-making and easing participation in EOL HIV cure research. Ensuring transparency about procedures, timelines, and safeguards against discomfort is essential for building trust and addressing lingering hesitations [7,9,20,23].

Beyond personal concerns, financial constraints shaped participants’ willingness to engage in EOL research. Many LTS with HIV faced significant economic insecurity, with burial and cremation costs adding to their financial strain. As their final days approached, these expenses created stress, and for some, the opportunity to participate in EOL research offered relief from this burden. Prior research on terminal illness and cancer trials shows financial instability can influence EOL decisions, with some viewing research as a way to lessen financial burdens on their next-of-kin [24]. The Last Gift study underscored the complexity of addressing EOL costs in research [13]. Concerns over undue influence resulted in initial hesitance from the IRB regarding the coverage of cremation or burial expenses, fearing it might pressure participation. However, after discussions with the community, researchers determined that this coverage was fair compensation for the participant’s time and this study’s invasiveness rather than an incentive [13]. To protect autonomy, the study team must ensure that body disposition is not framed as a benefit and implement measures to mitigate undue influence. Ethical EOL research must balance financial relief with participant autonomy [8]. While covering burial and cremation costs can ease stress, it must not become a coercive incentive. Ethical financial support, such as nonprofit partnerships or structured assistance programs, may alleviate monetary hardships, but integrating such efforts into research may burden study teams. Broader policy changes or external support networks may offer more sustainable solutions while preserving ethical integrity.

Geographic barriers also emerged as a significant limitation, with many participants expressing disappointment that EOL HIV cure research remained concentrated in select academic centers. They voiced a strong desire for the expansion of this type of research at other appropriate research centers across the U.S. Their recommendations mirror broader disparities in accessibility to HIV clinical trials, where PLWH in rural and underserved areas often face greater logistical burdens and fewer opportunities to participate. Building capacity for EOL research requires not only logistical readiness, such as timely autopsy protocols and regulatory processes, but also sustained community engagement and culturally competent research teams. Collaborating with rapid autopsy centers conducting EOL research in oncology could help scale efforts efficiently while sharing expertise and resources. Additionally, establishing regional centers of excellence across the U.S. could reduce geographic barriers and improve participation equity. Addressing logistical barriers through policy advocacy and structural interventions is critical. Providing transportation support, streamlining post-mortem logistics, and ensuring that participation does not impose financial burdens will create a more equitable EOL HIV research landscape. Expanding outreach beyond traditional HIV research networks through peer networks, LGBTQ+ centers, CBOs, social media, and multilingual materials can further improve engagement and accessibility.

Importantly, participants emphasized the need for ethical and rigorous EOL HIV cure research, highlighting concerns about past research abuses and a lack of representation, particularly among racial and ethnic minorities. Studies have shown that PLWH from marginalized communities are less likely to participate in clinical research due to historical mistreatment and a lack of culturally tailored engagement initiatives [25,26]. Addressing these issues requires intentional efforts to involve community leaders and peer advocates in outreach and education efforts. Additionally, gender and cultural factors played a role in shaping attitudes toward EOL research participation. Some participants, particularly women and individuals from religious backgrounds that emphasize bodily integrity, expressed concerns about rapid research autopsy procedures, burial practices, and personal agency in EOL decision making. Similar findings have been reported in studies on organ donation and post-mortem research participation, where religious and cultural beliefs significantly influence willingness to participate in research [27,28]. Research teams must accommodate religious and cultural considerations, offer flexible participation options, and ensure that research protocols respect diverse EOL preferences to create an inclusive and ethical research environment [8].

Concerns about hospice and palliative care access influenced LTS’s views on EOL HIV cure research. Many were unsure how study involvement might affect their supportive care, underlining the broader challenge of integrating research within long-term care settings [29]. While the Last Gift study operates independently of hospice and palliative care [13], findings from our study underscore the need for better coordination between research and EOL services. Disparities in EOL care are further compounded by aging, HIV stigma, financial insecurity, and systemic barriers, making it critical to adopt an equity and intersectionality-informed approach. This framework can help ensure that recruitment, care integration, and support services address the unique needs of marginalized populations. One means of accomplishing this would be to integrate HIV-informed palliative care specialists into the recruitment and informed consent processes to provide holistic support while safeguarding autonomy. Research on the dual role of clinician–researchers suggests that separating medical care from research discussions helps prevent conflicts of interest and undue influence [20]. Palliative care specialists could facilitate these conversations, ensuring participants receive unbiased information while making informed decisions.

Table 3 summarizes recommendations suggested by diverse Long-Term Survivors of HIV on Cure Research at the end of life across the U.S.

### Limitations

We acknowledge several limitations in this qualitative interview study. First, this study interviewed LTS who were willing to discuss EOL and research topics and had access to computer teleconference technology, which may have introduced selection bias. Particularly notable is the extensive level of prior involvement in HIV research and advocacy. The perspectives of PLWH who have not previously participated in research may differ, and this further highlights the need for ongoing efforts for outreach and engagement of diverse communities and new partnerships. Despite recruiting participants from across the U.S. and encompassing a diverse range of racial and ethnic backgrounds, Hispanic individuals were notably absent from this study, reflecting the ongoing underrepresentation of Spanish-speaking PLWH in HIV cure research in the U.S. Additionally, since both this study and its participants were based in the U.S., we acknowledge that our findings may not be generalizable to other contexts.

## 5. Conclusions

Research on HIV cure at EOL offers a unique opportunity to advance understanding of HIV reservoirs, but its success depends on addressing key social, ethical, and logistical considerations. The present study highlights the perspectives of diverse LTS of HIV across the U.S., whose willingness to participate is shaped by motivations like altruism and legacy, alongside barriers such as financial and invasiveness concerns, trust in science, and logistical challenges. While current EOL HIV cure studies focus on PLWH near the EOL, expanding research to include LTS in long-term planning could enhance engagement and equity. Transparent communication and culturally competent outreach are essential to building trust and inclusiveness. To ensure EOL cure research is ethically sound, accessible, and representative, future regulatory and policy considerations and community engagement strategies must align with the priorities of PLWH.

## Figures and Tables

**Table 1:** IRB-Approved Interview Guide: Perceptions of Diverse Long-Term Survivors of HIV on Cure Research at the end of life (The United States 2023–2024).

First, thank you so much for your time to participate in this interview about HIV cure-related research at the end of life, which we are now recording.
*Knowledge of HIV Cure-Related Research at the EOL*
In this part of our conversation, we will focus specifically on research conducted at the end of a participant’s life that is designed to help researchers develop strategies that may someday help cure other people with HIV. As you may know, there are various ways that community members support HIV-related clinical research. Some people serve on Community Advisory Boards, while others might respond to surveys, participate in focus groups, or even volunteer to participate in a clinical trial of an experimental therapy or other intervention.
In general, what do you think of research towards an HIV cure? Would you please describe any personal involvement you may have had up until today in HIV cure-related research? Have you heard of HIV cure-related research at the end of life?
*Perceptions of HIV Cure-Related Research at the EOL*
Research on HIV persistence—including HIV cure-related research at the EOL—represents a strategic research priority. The Last Gift study is one example of this type of research. The Last Gift is a clinical research study conducted at the University of California, San Diego, for PLWH who have a terminal illness such as cancer, advanced heart disease, or neurodegenerative disease like ALS [amyotrophic lateral sclerosis], often called Lou Gehrig’s Disease.
Do you think this research should be conducted [or not]? Why do you think/feel that way? Please explain. What concerns, if any, do you have about this sort of research? Do you think people with HIV would be generally interested in participating in HIV cure-related research at the EOL? What do you think would motivate people with HIV to participate in HIV cure-related research at the EOL? What do you think would deter people with HIV from participating in HIV cure-related research at the EOL? What do you think might be some of the concerns people with HIV have about participating in HIV cure-related research at the EOL? What kinds of support do you think would be needed to help people with HIV participate in HIV cure-related research at the EOL? Do you think people with HIV at the EOL would be willing to undergo invasive study procedures such as blood draws, leukaphereses, hair donations, gut biopsies, lymph node biopsies, etc.? Why or why not?
*Justice, Equity, and Trustworthiness Considerations*
What are some of the most effective ways to engage different groups in HIV cure research? What are some ways we should try to increase medical trust as it relates to HIV cure research at the EOL? What could be some of the cultural considerations for implementing HIV cure research at the EOL? What might be some religious or spiritual considerations for implementing HIV cure research at the EOL?
*Regulatory or Policy Considerations for HIV Cure-Related Research at the End of Life*
What should be some of the key policy priorities for conducting HIV cure-related research at the EOL?
*Closing*
Is there anything else you would like us to know about HIV cure-related research at the EOL?

**Table 2:** Demographic Characteristics of Long-Term Survivors of HIV on Cure Research at the end of life (The United States, 2023–2024).

Characteristics	N (%)
Age in years, mean (min–max)	68.4 (60–82)
Sex assigned at birth	
Male	9 (56.25)
Female	7 (43.75)
Gender	
Cisgender Man	9 (56.25)
Cisgender Woman	7 (43.75)
Education	
Less than high school	1 (6.25)
High school diploma or GED	1 (6.25)
Some college, but no diploma	4 (25)
College graduate	9 (56.25)
Master’s degree or equivalent	1 (6.25)
Race	
White/Caucasian	9 (56.25)
Black African/American	4 (25)
Asian	2 (12.5)
Mixed	1 (6.25)
U.S. Region	
Northeast	2 (12.5)
Midwest	1 (6.25)
South	5 (31.25)
West	8 (50)
Years worked in HIV-related advocacy in general, mean (std. dev.)	27.13 (10.46)
Years worked in HIV Cure-related advocacy, mean (std. dev.)	7.34 (9.78)

**Table 3:** Summary of Recommendations Suggested by Diverse Long-Term Survivors of HIV on Cure Research at the end of life. (United States, 2023–2024).

Key Considerations	Actionable Strategies
**Expanding Awareness and Early Engagement**	Broaden outreach to include LTS, considering future participation, not only those at the EOL. Utilize community-driven education, peer advocates, and community ambassadors to engage diverse populations. Expand the scope of outreach beyond traditional research centers to LGBTQ+ centers, CBOs, and intergenerational forums. Provide clear, accessible channels (e.g., email, institutional WhatsApp, phone line) for questions and researcher contact.
**Balancing Altruism with Ethical and Logistical Concerns**	Clearly communicate post-mortem logistics and provide ethical financial support options (e.g., nonprofit partnerships) to address burial and cremation concerns while avoiding undue influence. Offer transportation assistance and logistical support, especially for rural participants, while ensuring clarity on post-mortem procedures and burial timelines.
**Ethical and Inclusive Recruitment Practices**	Integrate HIV-informed palliative care specialists into recruitment to provide unbiased, holistic support. Use accessible educational materials (e.g., videos, diagrams) to improve comprehension. Strengthen ethics training for researchers and IRBs to prevent undue influence and ensure informed consent.
**Integrating Cultural and Religious Considerations**	Engage faith leaders and spiritual advisors to address religious concerns about body integrity. Ensure culturally competent study materials and diverse research teams. Develop alternative participation pathways that respect cultural traditions and accommodate religious rites where necessary.
**Providing Mental Health and Social Support**	Provide trauma-informed counseling and mental health resources to address anxieties around EOL research. Implement a study buddy peer support model for guidance and emotional support. Establish a hotline or on-call system for participants and their next-of-kin/loved ones.
**Ensuring Participant Comfort**	Prioritize pain management and comfort measures while acknowledging that some participants may have concerns about biopsies and post-mortem tissue retrieval.
**Expanding EOL HIV Cure Research Infrastructure**	Develop regional Centers of Excellence to expand access beyond major metropolitan areas. Partner with oncology rapid autopsy programs and hospice organizations to integrate EOL research into existing services.
**Strengthening Trust and Community Engagement**	Address historical medical distrust by ensuring transparent, community-led engagement efforts. Engage trusted community figures and peer advocates. Provide regular updates on research progress and outcomes to maintain transparency.
**Regulatory, Policy, and Structural Support**	Ensure sustained policy support, ethical oversight, and stable funding to maintain long-term feasibility and equitable access to EOL HIV cure research.

LTS: Long term Survivors; EOL: End of Life; CBO: Community-based organizations; IRB: Institutional Review Board; LGBTQ+: Lesbian, Gay, Bisexual, Transgender, and Queer or Questioning.

## Data Availability

The original contributions presented in this study are included in the article/Supplementary Materials. Further inquiries can be directed to the corresponding author.

## Supplementary Materials

The following supporting information can be downloaded at: https://www.mdpi.com/article/10.3390/idr17040078/s1, Table S1: Additional Quotes—Perceptions of Long-Term Survivors of HIV about End-of-Life HIV Cure Research (United States, 2023–2024).

## References

[1] Harper J., Betts M.R., Lichterfeld M., Müller-Trutwin M., Margolis D., Bar K.J., Li J.Z., McCune J.M., Lewin S.R., Kulpa D. (2023). Progress Note 2024: Curing HIV; Not in My Lifetime or Just Around the Corner?. Pathog. Immun..

[2] Alum E.U., Uti D.E., Ugwu O.P., Alum B.N. (2024). Toward a cure—Advancing HIV/AIDs treatment modalities beyond antiretroviral therapy: A Review. Medicine.

[3] Deeks S.G., Archin N., Cannon P., Collins S., Jones R.B., de Jong M., Lambotte O., Lamplough R., Ndung’u T., Sugarman J. (2021). Research priorities for an HIV cure: International AIDS Society Global Scientific Strategy 2021. Nat. Med..

[4] Rawlings S.A., Layman L., Smith D., Scott B., Ignacio C., Porrachia M., Concha-Garcia S., Hendrickx S., Kaytes A., Taylor J. (2020). Performing rapid autopsy for the interrogation of HIV reservoirs. AIDS.

[5] UCSD (2017). University of California San Diego; The Last Gift Study: Advancing HIV Cure Research at End of Life. UC San Diego. https://lastgift.ucsd.edu/.

[6] The Well Project: Long-Term Survivors of HIV (2023). The Well Project. https://www.thewellproject.org/hiv-information/long-term-survivors-hiv.

[7] Ndukwe S.O., Patel H., Shelton B., Concha-Garcia S., Dullano C., Solso S., Hendrickx S., Riggs P.K., Villa T.J., Kaytes A. (2024). People with HIV at the end-of-life and their next-of-kin/loved ones are willing to participate in interventional HIV cure-related research. AIDS.

[8] Kanazawa J., Gianella S., Concha-Garcia S., Taylor J., Kaytes A., Christensen C., Patel H., Ndukwe S., Rawlings S.A., Hendrickx S. (2022). Ethical and practical considerations for HIV cure-related research at the end-of-life: A qualitative interview and focus group study in the United States. BMC Med. Ethics.

[9] Dubé K., Villa T.J., Taylor J., Kaytes A., Moore D.J., Little S.J., Chaillon A., Smith D.M., Gianella S. (2023). A Community-Driven Framework to Prioritize the Use of Donated Human Biological Materials in the Context of HIV Cure-Related Research at the End of Life. Pathog. Immun..

[10] Dubé K., Shelton B., Patel H., Ndukwe S.O., Concha-Garcia S., Dullano C., Solso S., Hendrickx S., Kaytes A., Taylor J. (2023). Perceived risks and benefits of enrolling people with HIV at the end of life in cure research in Southern California, United States. J. Virus Erad..

[11] Cosentino F., Marino A., Anile L., Moscatt V., Gussio M., Boscia V., Bruno R., Nunnari G., Pulvirenti A., Privitera G.F. (2023). Long-Term Survivors in a Cohort of People Living with HIV Diagnosed between 1985 and 1994: Predictive Factors Associated with More than 25 Years of Survival. Infect. Dis. Rep..

[12] Dācus J.D., Wood C.V., Ward D. (2023). Partnering and Programming for a Black, Indigenous, and People of Color Sexual and Gender Minority Pathway to HIV Research. J. Acquir. Immune Defic. Syndr..

[13] Kanazawa J., Rawlings S.A., Hendrickx S., Gianella S., Concha-Garcia S., Taylor J., Kaytes A., Patel H., Ndukwe S., Little S.J. (2023). Lessons learned from the Last Gift study: Ethical and practical challenges faced while conducting HIV cure-related research at the end of life. J. Med. Ethics.

[14] Creswell J.W. (2007). Qualitative Inquiry and Research Design: Choosing Among Five Approaches.

[15] Guest G., Bunce A., Johnson L. (2006). How Many Interviews Are Enough?: An Experiment with Data Saturation and Variability. Field Methods.

[16] Hsieh H.F., Shannon S.E. (2005). Three approaches to qualitative content analysis. Qual. Health Res..

[17] Bloomer M.J., Hutchinson A.M., Brooks L., Botti M. (2018). Dying persons’ perspectives on, or experiences of, participating in research: An integrative review. Palliat. Med..

[18] Javadi S.S., Mathur K., Concha-Garcia S., Patel H., Perry K.E., Lo M., Taylor J., Kaytes A., Little S., Gianella S. (2021). Attitudes and perceptions of next-of-kin/loved ones toward end-of-life HIV cure-related research: A qualitative focus group study in Southern California. PLoS ONE.

[19] Meanley S., Rodriguez Garcia L., Lisha N.E., Ahmed A., Korolkova A., Figueroa T., Nguyen E., Peluso M.J., Cohn L.B., Deeks S. (2025). Exploring Stigma and Self-Image: Mixed-Methods Insights from HIV Cure-Related Research Participants Undergoing Analytical Treatment Interruptions. AIDS Patient Care STDS.

[20] Deiss R., Gianella S., Dullano C., Solso S., Little S.J., Kaytes A., Taylor J., Riggs P.K., Hastie E., Smith D. (2025). Navigating the dual role of physician and clinician investigator in end-of-life research. AIDS Care.

[21] Hooper J.E. (2021). Rapid Autopsy Programs and Research Support: The Pre- and Post-COVID-19 Environments. AJSP Rev. Rep..

[22] Tutty E., Horsley P., Forbes Shepherd R., Forrest L.E. (2021). The art and science of recruitment to a cancer rapid autopsy programme: A qualitative study exploring patient and clinician experiences. Palliat. Med..

[23] Lindell K.O., Erlen J.A., Kaminski N. (2006). Lessons from our patients: Development of a warm autopsy program. PLoS Med..

[24] Baker K.M., Andersen L., McHugh M., Foxwell A.M., Zhou Q., Ratcliffe S.J., Huang L., Aryal S., Grady C., Ulrich C.M. (2025). Regret in Clinical Trial Participation Among Cancer Patients. J. Palliat. Med..

[25] Rao E., Taylor J., Kaytes A., Concha-Garcia S., Riggs P.K., Smith D.M., Dubé K., Gianella S. (2024). Vulnerability in Biomedical Research: A Historical Reflection and Practical Implications for HIV Cure-Related Research. AIDS Res. Hum. Retroviruses.

[26] Roberts C., Creamer E., Boone C.A., Young A.T., Magnus M. (2022). Short Communication: Population Representation in HIV Cure Research: A Review of Diversity Within HIV Cure Studies Based in the United States. AIDS Res. Hum. Retroviruses.

[27] Carola V., Morale C., Vincenzo C., Cecchi V., Errico L., Nicolais G. (2023). Organ donation: Psychosocial factors of the decision-making process. Front. Psychol..

[28] Crouch E.E., Damas C., Bartrug W.C., Shamiyeh A., Scelfo M., Dreyfus M., Gano D., Segal S., Franck L.S. (2023). Parents’ Views on Autopsy, Organ Donation, and Research Donation After Neonatal Death. JAMA Netw. Open.

[29] Pantilat S.Z., O’Riordan D.L., Dibble S.L., Landefeld C.S. (2010). Hospital-based palliative medicine consultation: A randomized controlled trial. Arch. Intern. Med..

