# Supplementary material for: “This Is How I Give Back”: Long-Term Survivors on Legacy and HIV Cure Research at the End of Life—A Qualitative Inquiry in the United States
Source: Infect Dis Rep. 2025 Jul 4;17(4):78. doi: 10.3390/idr17040078 (PMC12286158; doi:10.3390/idr17040078)
Supplement: Supplementary file 1 [file idr-17-00078-s001.zip › idr-3611761-supplementary.pdf]

Supplementary Table S1: Additional Quotes – Perceptions of Long-Term Survivors of HIV about End-of-Life HIV Cure Research (United States, 2023 – 2024)

Themes	Participants	Quotations
**Awareness and Perceptions of HIV Cure Research at the EOL**		
Awareness of HIV Cure Research at the EOL	Cisgender Man, White	*I know, Last Gift was a pioneering program. I discovered this research [EOL] exists because I watched a video. More people need to see that video… more people will participate.*
	Cisgender Man, White	*Sure. Well, I specifically heard about the Last Gift project, and I had been asked to work with [a researcher] at NIH to set up similar projects in the [East Coast] area.*
	Cisgender Man, White	*Oh, I think it's a wonderful thing. You know, the video [Last Gift study] shown in the webinar was so well done, and so powerful that if I hadn't already wanted to be in this study, I would have said, Sign me up.*
	Cisgender Female, White	*Actually, the only thing I've heard about was Last Gift, which somebody mentioned to me on a walk. Somebody from my aging with HIV group described it as being autopsied almost immediately after death to find hidden reservoirs of HIV. That sounded really compelling, but I've not heard about other things.*
	Cisgender Female, White	*Even though I live in [West], and I'm part of that community, it's just been recently that I discovered the ACTG [Advancing Clinical Therapeutics Globally]. And it's kind of shameful that the word isn't getting out to more people. I never had a doctor mention it to me.*
	Cisgender Male, Asian/Indian	*I thought I saw an interview about this on a mailing list, which was useful. But this should be more on YouTube, so if someone types 'HIV' it comes up immediately.*
	Cisgender Man, White	*If you want me, I'll be an ambassador for your last-of-life [Last Gift] thing. I'll go to places and talk about it… This is how you get people interested.*
	Cisgender Male, Asian/Indian	*Education is one big part. I found that a lot of people, even in the gay community, don't really know what AIDS is anymore. We need to talk to people and bring them into conversation of HIV Cure research.*
	Cisgender Man, White	*Let's have a symposium at the gay center on HIV and how to talk about it. Get the young and the old together in the same room.*
Perceptions of HIV Cure Research at the EOL	Cisgender Man, White	*To me, it's a real validation. After living with HIV for more than half my life, being able to make a final gesture… I mean, you gotta go in knowing it's not going to help you. But it's a wonderful opportunity for both us and the scientific community.*
	Cisgender Female, White	*If a trial could figure that out, you could be taken care of before it came to you.*
	Cisgender Male, White	*It's a fascinating study. The fact that you're collecting tissues and finding where reservoirs are—where the virus is latent—is really cool.*
	Cisgender Female, Mixed race	*Because there's a big world out there, and a lot of HIV, and a lot to be learned. We're in kindergarten right now, we'll figure it out.*
	Cisgender Male, White	*I think it's probably necessary to lead to a cure. So yes, it should be done, if you can manage to get it done.*
	Cisgender Female, Asian	*I think it should be done because no matter how much we know about HIV; there's always something to learn.*
**Motivations and Barriers for Participation in HIV Cure Research at the EOL**		
Altruism versus Fear of Discomfort and the Unknown	Cisgender Female, White	*People who have been living with HIV for a long time would really like to contribute in some meaningful way.*
	Cisgender Man, White	*If people think they'll be exposed to great discomfort or pain, that might be a deterrent. You need to have an honest presentation of what's involved.*
Desire for Connection and Dignity versus Family and Social Pressure	Cisgender Man, White	*There's something about being in a study where you're in a supportive group of people who are caring about you, who you can talk to.*
	Cisgender Female, Black	*Some people just don't want their family member's body cut up or taken away.*
Practical Considerations versus Logistical Barriers	Cisgender Man, White	*The cheapest cremation I could find was over $4,000. That's a lot of money. Some people just don't have that.*
	Cisgender Female, Black	*Money is the first thing that comes to mind. Funeral costs, transportation, living expenses... it all adds up.*
	Cisgender Female, Mixed race	*[Research teams would need to provide] their transportation costs… if it's a ride, or a ride for them and their caregivers… that trips to participate … shouldn't be a barrier.*

	Cisgender Female, Asian	*How long do they keep the body before returning it to the family? Some studies keep the body for a whole year. That would be a problem*
**Willingness to Undergo Invasive Study Procedures near or at the EOL**		
Willingness to Undergo Invasive Study Procedures near or at the EOL	Cisgender Man, White	*As long as it's done carefully, and pain management is a part of it, I don't see it as a deterrent.*
	Cisgender Male, White	*If I've got a form of cancer keeping me in constant pain, and you're going to add to it by doing a gut biopsy, do I really want to do that?*
	Cisgender Female, Asian	*Yeah, I'd like to know about it before I know that I'm gonna die. I think it's like, it's good to know about it, because you can talk to other people that are going into it. Yeah. Yeah. For myself, you know, I, when I know something, I share it to my community.*
	Cisgender Man, White	*I think they'd be just as willing to do it then as they wouldn't, you know, other times in their life. I'm not sure what leukapheresis is, maybe you can tell me?*
	Cisgender Man, White	*A medical professional should be the one to advise on these kinds of procedures.*
	Cisgender Male, White	*If the wins are talked about enough and people understand the value and potential, I think it's easier to be willing.*
	Cisgender Male, Black	*Are they going to give me something to numb me, so I don't really feel it? I don't think people are too anxious to have pain in the name of science.*
	Cisgender Male, White	*It is really incumbent on the study to make sure that the informed consent fully explains, even with diagrams, what these things are.*
**Outreach, Justice, Equity and Trustworthiness Considerations for HIV Cure Research at the EOL**		
Ensuring Transparency and Informed Decision-Making	Cisgender Female, Black	*Lack of knowledge is a big deterrent. If a person isn't informed about what the research is, they might be reluctant to be a part of it.*
	Cisgender Male, Black	*The more clear [clearer] information about the program, the more it will convince someone to participate.*
	Cisgender Female, White	*First of all, make the information widely available. Answer a lot of questions ahead of time because people are going to have a lot of questions about this sort of thing.*
	Cisgender Male, Black	*Be upfront. Be honest and clear about what your intentions are. People are untrusting these days, because of obvious reasons. So the more upfront you're going to be, and your clarity and sincerity, that's more [important].*
	Cisgender Female, Asian	*Having someone talk to the families, to ease their mind that their body will be used, but in a respectful way.*
	Cisgender Female, Black	*Talking to the people, yeah, giving them more information about this research. That's why I say the top key to this is communication… If they don't know about it, they're not going to do it.*
Addressing Mental Health and Social Support Needs	Cisgender Man, White	*I think that more attention needs to be drawn to it, especially with dementia, memory care, and other comorbidities with HIV.*
Peer and Community Support	Cisgender Male, White	*For cure-related research, people will need serious support—someone to help take care of them, get them places, keep an eye on them.*
Expanding Outreach Beyond Traditional Research Centers	Cisgender Man, White	*People are not going to come to you. You need to go to them, community centers, pride events, even small towns in rural areas.*
Reducing Stigma Through Representation and Inclusive Research	Cisgender Man, White	*The only way we can embrace differences is to... be inclusive in our outreach and personnel.*
	Cisgender Female, White	*People need to hear from people like you. A good speaker, someone they trust. That's how you get people interested.*
Tailored Communication and Accessibility	Cisgender Male, White	*The people approaching these folks need to be culturally competent. They need to be culturally aware. They need to approach things… without assumptions.*
Addressing Historical Distrust and Medical Mistrust	Cisgender Female, Mixed race	*It was something that came from the government, and people are just dissatisfied with what's happened in their life. Some don't trust the system.*
	Cisgender Male, White	*There are still people out there who believe Black people don't feel pain the same way white people do… The Tuskegee Experiment was 100 years ago, but the damage is still real.*
**Religious and Cultural Considerations in HIV Cure Research at End of Life**		
Religious and Spiritual Considerations	Cisgender Female, Black	*All Muslims don't like to be cut up. They purify the body, wrap them in sheets, and put them in the ground. No burning, no taking tissues or organs.*
	Cisgender Male, White	*Superstition? I'm sure there are certain segments of the population for whom this sort of thing breaks cultural boundaries or taboos.*
	Cisgender Man, White	*If you're there within six hours, and you made a deal with a rabbi, there may be some way to do it. And with Catholics, there may be some kind of secret blessings.*
	Cisgender Female, Black	*It would need to be tailored for each nationality and religion.*

	Cisgender Male, Asian	*There are religions where you have to be buried intact, and that could be a problem.*
	Cisgender Female, White	*Some religions believe in resurrection and want the body exactly as it was.*
	Cisgender Female, Black	*Muslims, Arabs, and Cambodians have different beliefs on what can be done to their bodies.*
Cultural and Gender Considerations	Cisgender Man, White	*I just look at my mother, who did not want to discuss her urinary incontinence with a man. I had to find a woman doctor. That was relatively easy to do. So, I think that there are differences, and the only way we can embrace those differences is to learn about the other groups of people.*
	Cisgender Man, White	*Mexican culture has the Day of the Dead. Jewish traditions involve sitting Shiva.*
**Regulatory and Policy Considerations of HIV Cure Research at the EOL**		
Autonomy, Hospice, and Equitable Care	Cisgender Male, Black	*Security, having shelter is the main thing. A lot of people don't have that, regardless of whether they're HIV positive or not.*
	Cisgender Female, Black	*I think I would want the responsibility of paying rent or mortgage lifted at the end of life. That should be one of the last things a dying person or their family has to worry about.*
	Cisgender Man, White	*If you live in a place where there is healthcare, but you can't afford it, it means nothing. You don't want people to go broke at the end of their life or their families to go broke trying to provide a nice death.*
	Cisgender Man, White	*There is a law in [Country], it's called the MAiD law. I don't know much about it... but obviously, their public policy does deal with assisted suicide, or end-of-life choices. And basically, they want to honor what that is.*
End-of-Life Planning and Legal Protections	Cisgender Female, Black	*Here in [City], [legal law school] helps with living wills, power of attorney… States that don't have that should make it widely available, especially for older people living with HIV.*

EOL: End of Life
